# Effects of exogenous pyoverdines on Fe availability and their impacts on Mn(II) oxidation by *Pseudomonas putida* GB-1

**DOI:** 10.3389/fmicb.2014.00301

**Published:** 2014-06-25

**Authors:** Sung-Woo Lee, Dorothy L. Parker, Kati Geszvain, Bradley M. Tebo

**Affiliations:** ^1^Division of Environmental and Biomolecular Systems, Institute of Environmental Health, Oregon Health & Science UniversityPortland, OR, USA; ^2^Geosciences Research Division, Scripps Institution of Oceanography, University of California, San DiegoSan Diego, CA, USA

**Keywords:** pyoverdine, Fe availability, iron requirement, iron limitation, siderotyping, MnO_2_, pyoverdine receptor, biofilm

## Abstract

*Pseudomonas putida* GB-1 is a Mn(II)-oxidizing bacterium that produces pyoverdine-type siderophores (PVDs), which facilitate the uptake of Fe(III) but also influence MnO_2_ formation. Recently, a non-ribosomal peptide synthetase mutant that does not synthesize PVD was described. Here we identified a gene encoding the PVD_GB-1_ (PVD produced by strain GB-1) uptake receptor (PputGB1_4082) of strain GB-1 and confirmed its function by in-frame mutagenesis. Growth and other physiological responses of these two mutants and of wild type were compared during cultivation in the presence of three chemically distinct sets of PVDs (siderotypes n°1, n°2, and n°4) derived from various pseudomonads. Under iron-limiting conditions, Fe(III) complexes of various siderotype n°1 PVDs (including PVD_GB-1_) allowed growth of wild type and the synthetase mutant, but not the receptor mutant, confirming that iron uptake with any tested siderotype n°1 PVD depended on PputGB1_4082. Fe(III) complexes of a siderotype n°2 PVD were not utilized by any strain and strongly induced PVD synthesis. In contrast, Fe(III) complexes of siderotype n°4 PVDs promoted the growth of all three strains and did not induce PVD synthesis by the wild type, implying these complexes were utilized for iron uptake independent of PputGB1_4082. These differing properties of the three PVD types provided a way to differentiate between effects on MnO_2_ formation that resulted from iron limitation and others that required participation of the PVD_GB-1_ receptor. Specifically, MnO_2_ production was inhibited by siderotype n°1 but not n°4 PVDs indicating PVD synthesis or PputGB1_4082 involvement rather than iron-limitation caused the inhibition. In contrast, iron limitation was sufficient to explain the inhibition of Mn(II) oxidation by siderotype n°2 PVDs. Collectively, our results provide insight into how competition for iron via siderophores influences growth, iron nutrition and MnO_2_ formation in more complex environmental systems.

## Introduction

Manganese (III, IV) oxides are naturally abundant minerals that control the environmental fate of various organic compounds and metals through adsorption and redox processes (Tebo et al., 2004; Webb et al., 2005). Although manganese oxides can be produced via both biological and abiotic oxidation of Mn(II), biological oxidation of manganese has been reported to be orders of magnitude faster (Hastings and Emerson, 1986). One of the model organisms used to study bacterial oxidation of Mn(II) is *Pseudomonas putida* GB-1, a bacterium isolated from a freshwater environment (Okazaki et al., 1997; Brouwers et al., 1999; Murray et al., 2005; Toner et al., 2005).

In aerobic and neutral pH environments where most Mn(II)-oxidizing bacteria have been isolated, iron is mainly found as insoluble Fe(III) with limited bioavailability. To overcome iron limitation, fluorescent pseudomonads, which include *P. putida* GB-1, produce pyoverdines (PVDs), fluorescent siderophores with high affinity for Fe(III) (Parker et al., 2004). The PVDs produced by different *Pseudomonas* isolates generally share the same quinoleinic chromophore joined to a polypeptide chain that varies in length and composition from strain to strain (Hohnadel and Meyer, 1988). Fe(III) bound by a particular PVD enters the cell via a specific PVD-Fe(III) cognate receptor that ordinarily recognizes the polypeptide portion of only that PVD (Clement et al., 2004), although rarely a receptor may recognize more than one PVD (Meyer et al., 1999, 2002b) or a strain may produce a receptor for a PVD that it does not synthesize (Koster et al., 1995). The usual specificity of each PVD for a particular cognate receptor forms the basis of siderotyping, which assigns the PVDs produced by different *Pseudomonas* isolates into groups of identical isoelectric focusing properties and identical cellular uptake by reference strains (Fuchs et al., 2001; Meyer et al., 2002a).

It was previously shown that PVDs can form stable and soluble complexes of Mn(III) with high stability constants (logK) comparable or slightly higher than that for Fe(III), 47.5 and 44.6, respectively (Parker et al., 2004). In fact, the addition of purified homologous PVD to iron-replete cultures of *P. putida* GB-1 inhibited MnO_2_ formation, with the extent and duration of inhibition depending on the PVD concentration and correlating with the concentration of the PVD-Mn(III) complex (Parker et al., 2007). The PVD-Mn(III) complex was stable and MnO_2_ production was inhibited for >30 days at 25°C when the PVD concentration exceeded the sum of the Mn and Fe concentrations in the culture. If the PVD concentration was less than the Mn concentration, however, MnO_2_ formed slowly and the PVD was apparently degraded or modified, as indicated by altered absorption and fluorescence spectra (Parker et al., 2007). The formation of the PVD-Mn(III) complex did not require the presence of *P. putida* GB-1 cells [e.g., it was observed in sterile aerated medium containing only Mn(II) and purified PVD] and occurred with the Mn(II)-oxidation-deficient mutant GB-1-007 (Parker et al., 2007), suggesting a non-enzymatic mechanism resembling other ligand-promoted reactions of Fe(II) or Mn(II) with oxygen (Klewicki and Morgan, 1998; Xiao and Kisaalita, 1998; Duckworth and Sposito, 2005).

The studies above involved cultures of single strains, each challenged with PVD it synthesized. In the environment, however, various organisms can share the same habitat and experience the same environmental conditions, e.g., iron-limitation. In such cases, organisms that are capable of producing siderophores or other ligands will do so, exposing all community members to iron bound by diverse siderophores. In these situations, the ability to use available heterologous siderophores (siderophores different from ones the organism produces) becomes crucial in dictating the actual iron availability to a particular organism. Under these circumstances, “cheaters” (Griffin et al., 2004; Harrison et al., 2008; Harrison and Buckling, 2009) or “predators” (Meyer et al., 2002b) that can use heterologous siderophores could gain competitive advantage.

Recently, the PVDs produced by several Mn(II)-oxidizing pseudomonads have been siderotyped and placed into three groups, n°1, n°2, and n°4, with the most important PVD similarities being confirmed by MS/MS (Parker et al., 2014). Here we investigated the effects of these three PVD siderotypes on iron availability and MnO_2_ formation by *P. putida* GB-1, extending previous studies of the PVD-mediated inhibition of MnO_2_ production, which had all involved only the homologous PVD. Data for homologous PVDs are hard to interpret because homologous PVD could conceivably influence Mn(II) oxidation through at least two mechanisms: simple effects on iron availability and more complex regulatory phenomena involving interactions of homologous PVD-Fe(III) complexes with their cognate uptake receptor, which has been reported to signal PVD synthesis in *Pseudomonas aeruginosa* (Lamont et al., 2002). We begin to disentangle these two possibilities by utilizing PVDs of siderotypes n°2 and n°4, which differ in their receptor specificities from the PVD produced by strain GB-1 (siderotype n°1). Another goal of this study was to investigate how exposure to heterologous PVDs at various conditions influences the synthesis of homologous PVD, MnO_2_, and biofilms by strain GB-1, in an attempt to understand effects that could occur naturally within mixed microbial communities in iron-limited habitats.

## Methods and materials

### Media

To determine the effects of exogenous PVD on PVD synthesis and biofilm formation, succinate medium (Meyer et al., 1997) and MSGFeMn medium (Parker et al., 2007) were used, respectively. Succinate medium, pH 7, included (per L) 6 g K_2_HPO_4_, 3 g KH_2_PO_4_, 1 g (NH_4_)_2_SO_4_, 0.2 g MgSO_4_•7H_2_O, 4 g succinic acid with 2 μM FeSO_4_•7H_2_O or 2 μM FeCl_3_ with 10 μM PVD. PVD was added prior to the addition of freshly made (0.2 μm filtered) Fe solution to prevent precipitation of iron. MSGFeMn medium contained (per L) 0.24 g (NH_4_)_2_SO_4_, 0.06 g MgSO_4_•7H_2_O, 0.06 g CaCl_2_•2H_2_O, 0.02 g KH_2_PO_4_, 0.3 g Na_2_HPO_4_, 5 mM glucose, 2 μM FeSO_4_•7H_2_O 100 μM MnCl_2_, and was aseptically adjusted to pH 7.1 and then buffered with 50 mM HEPES (pH 7.5). To test the effect of exogenous PVD on the growth of an NRPS mutant of *P. putida* GB-1 (KG163, PputGB1_4083::pKG220, Gm^R^), CAA media supplemented with 100 μM 2, 2′-dipyridyl (Moon et al., 2008) was used. CAA medium (per L) consisted of 5 g casamino acid, 1.18 g K_2_HPO_4_•3H_2_O, and 0.25 g MgSO_4_•7H_2_O. The Leptothrix (Lept) agar used for co-cultivation experiments (Boogerd and de Vrind, 1987) consisted of 0.5 g yeast extract, 0.5 g casamino acids, 5 mM D(+)-glucose, 10 mM HEPES (*N*-2-hydroxyethylpiperazine-*N*'-2-ethanesulfonic acid), pH 7.5., 0.48 mM CaCl_2_, 0.83 mM MgSO_4_, 3.7 μM FeCl_3_, 100 μM MnCl_2_, 1.5% Noble agar and 1 mL of a trace element solution containing (per L) 10 mg CuSO_4_•5H_2_O, 44 mg ZnSO_4_•7H_2_O, 20 mg CoCl_2_•6H_2_O, and 13 mg Na_2_MoO_4_•2H_2_O.

### MnO_2_ formation during co-growth of *p. putida* GB-1 with various PVD producers

**Figure 1:**
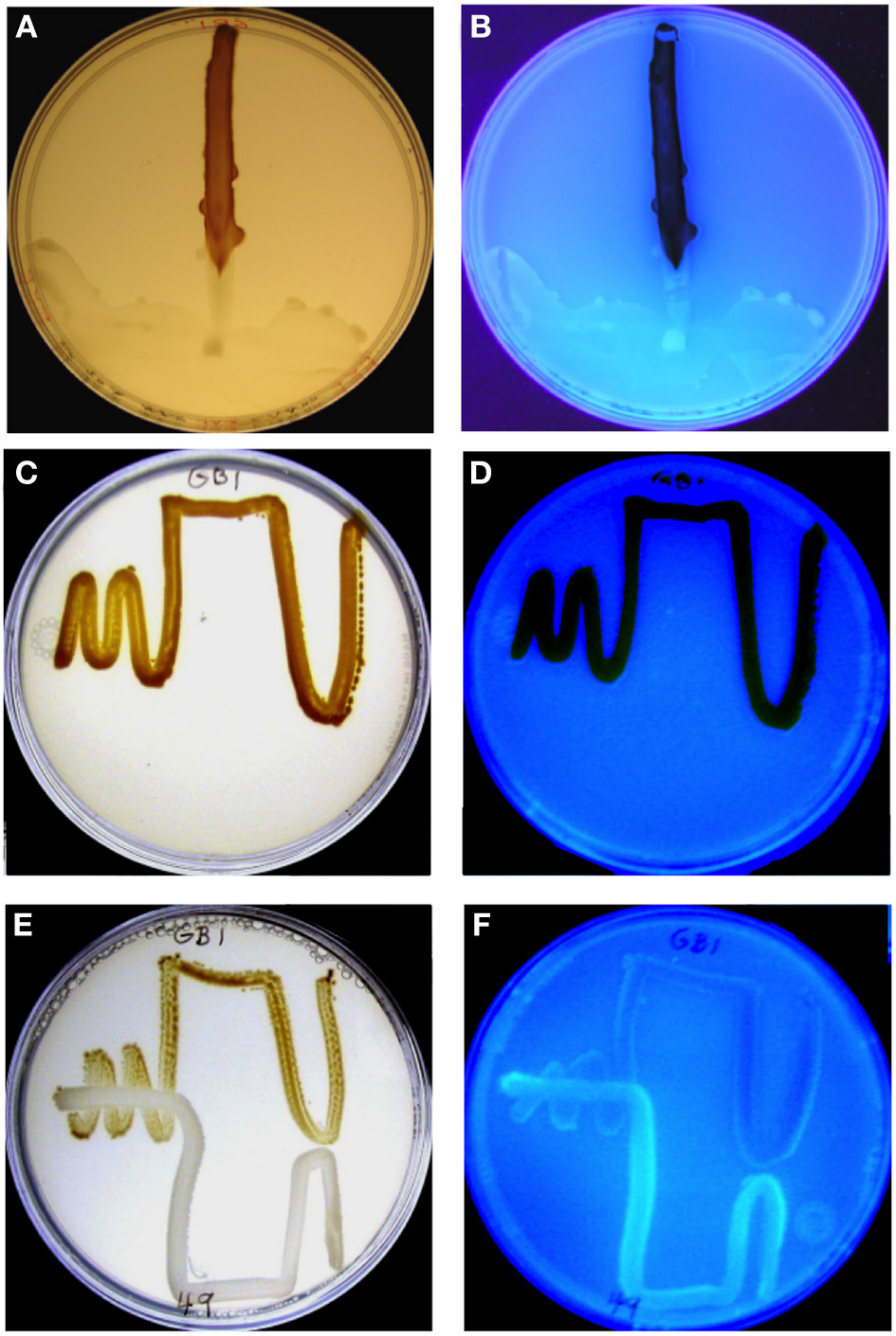
**MnO_2_ formation by *P. putida* GB-1 after exposure to purified PVD_GB-1_ (A,B) and to PVD released during the growth of another siderotype n°1 strain, *P. putida* CFML90-49 (E,F). (A,C,E)** Visible light illumination to show brown MnO_2_. **(B,D,F)** UV light illumination to detect blue-green PVD fluorescence. **(A,B)** Effect of 75 μL of purified PVD_GB-1_ (200 mM, siderotype n°1) applied at one edge of a plate containing 20 ml of solid medium and a single streak of *P. putida* GB-1. The plate was incubated vertically so that the liquid remained localized, although it did spread as a crescent at the edge of the plate on the downward side, taking some inoculum with it. Note that areas of the strain GB-1 streak line near the zone of PVD_GB-1_ application showed considerable blue-green PVD fluorescence, whereas growth zones further from the PVD drop contained brown MnO_2_ with no visible fluorescence. **(C,D)** Full MnO_2_ production when strain GB-1 was cultured alone. **(E,F)** Inhibited MnO_2_ production when strain GB-1 was co-cultivated with *P. putida* CFML90-49, which cannot oxidize Mn(II) and produces a siderotype n°1 PVD resembling PVD_GB-1_. Note the increased PVD fluorescence and decreased MnO_2_ within strain GB-1 growth zones in the presence of strain CFML90-49 **(E,F)** as compared to the control without strain CFML90-49 **(C,D)**.

Leptothrix (Lept) agar, supplemented with 2 μM FeCl_3_ from a freshly-prepared and filtered (0.2 μm pore size) stock of 10 mM FeCl_3_, was poured in precise 20 mL portions into 100 × 15 mm plastic Petri plates. Before inoculation, all plates were placed in a sealed bag and aseptically stored for >1 week at 4°C to allow the chemical speciation of iron to equilibrate so that siderophore synthesis was reproducible in inoculated plates. Replicate plates (duplicate, triplicate, or quintuplicate in three replicate experiments) were inoculated from overnight cultures grown in liquid Lept medium (Boogerd and de Vrind, 1987). In the two initial experiments, the test organism (*P. putida* GB-1) was streaked in one direction and then a challenge strain (a PVD-producing pseudomonad or a control that could not make PVD) was streaked at right angles to it (cross-streak plates). In the third experiment, a somewhat different streaking geometry was used (see Figures 1C–F). Duplicate control plates were inoculated with each organism alone. All plates were sealed with parafilm, inverted, and incubated at 23–25°C in the dark. At 1, 2, 3, 4, 5, and 10 days of incubation, experimental and control plates were illuminated from the top and side by Silvania F40W/SS fluorescent lights and photographed with the Dimage X60 camera (JPEG; red/green/blue; 2560 × 1920 pixels). Results were scored as inhibition of MnO_2_ formation if either of the following was observed: (1) a pronounced gradient of decreased MnO_2_ density beginning near the challenge strain and changing with distance from it or (2) a difference of at least 3-fold in the average density of MnO_2_ (measured on photographs) in a cross streak plate vs. the appropriate controls with test organism only. Table 1 presents the pooled data for experiments 1 and 2 and combines the results observed for each plate at 3, 4, 5, and 10 days, since the scored result did not change after 3 days. Photographs in Figure 1 were taken at day 4 of experiment 3.

**Table 1:** **Effect of PVD-producing organisms with various siderotypes on MnO_2_ formation by *P. putida* GB-1 (siderotype n°1) during co-cultivation conditions**.

**Challenge organism**		**Siderotype^b^**	**Effect on MnO_2_ formation by *P. putida* GB-1 (PVD n°1)^c^**		
**Strain**	**Forms MnO_2_?^a^**				
MnB1	Slow	1	I (2/2)		
CFML90-45	No	1	I (2/2)		
CFML90-48	No	1	I (1/2),		N(1/2)
CFML90-49	No	1	I (2/2)		
CFML90-50	No	1	I (2/2)		
CFML90-51	No	1	I (4/5),		N (1/5)
KT2440	At low O_2_ only	2	I (3/5),		N (2/5)
ISO6	Slow	4	N (5/5)		

a *Qualitative rate of MnO_2_ production is reported relative to that by P. putida GB-1*.

b *Determined previously (Parker et al., 2014)*.

c *I, inhibition of MnO_2_ formation; S, stimulation of MnO_2_ formation; N, no detectable effect. Effects were read, in comparison to controls with strain GB-1 only, at 4 days of growth at 23–25°C, but results at 5 and 10 days were the same as those at 4 days. Results were confirmed from photographs (examples in Figure 1) taken at multiple times during growth. Numbers in parentheses indicate ratio of Petri plates that showed the reported result among the total number of plates tested*.

### Purification of PVD

*P. putida* GB-1, *P. putida* CFML 90-51, *P. putida* KT2440, *Pseudomonas* sp. PCP1, and *Pseudomonas* sp. ISO6 were grown in CAA media at room temperature. Cultures were centrifuged, filtered, and then adjusted to pH 5.5 with HNO_3_. PVD was purified using previously reported methods (Parker et al., 2014). The filtrates were run through SepPak C18 columns, washed with 5 volumes of Milli-Q deionized water (18.2 MΩ), eluted with 50% methanol in Milli-Q water, lyophilized and resuspended in Milli-Q water.

### Effect of exogenous PVDs on growth of a PVD synthesis mutant of strain GB-1

A non-ribosomal peptide synthetase (NRPS) mutant was created previously with a plasmid integration into PputGB1_4083, which was shown to be the NRPS for synthesis of the peptide backbone of PVD_GB-1_ (Parker et al., 2014). This mutant is deficient in PVD synthesis (Parker et al., 2014). The mutant was grown overnight in LB medium supplemented with 50 μgml^−1^ gentamicin. It was subcultured in LB broth and grown until it reached mid-exponential phase, at which point it was spread onto a CAA plate amended with 100 μM dipyridyl (Moon et al., 2008). Filtered disks soaked with 20 μl of various 1 mM PVDs were then put on top of the overlay to check for growth inhibition around the disks.

### In-frame deletion of a gene encoding the putative receptor of PVD_GB-1_

In-frame deletion of the putative ferri-PVD_GB-1_ receptor gene PputGB1_4082 followed previously described methods (Geszvain and Tebo, 2010). Table 2 lists the primers used to amplify ~500 bp regions upstream and downstream of the targeted gene PputGB1_4082. These regions were then fused. Strains and plasmids used in this study are listed in Table 3. To determine whether the target gene was indeed deleted, genomic DNA was extracted from the candidate strains using a commercially available kit (Wizard® Genomic DNA isolation system, Promega), and PCR was then performed using primers 4081F and 4083R (Table 2).

**Table 2:** **Primers used for in-frame deletion of putative ferri-PVD receptor, PputGB1_4082**.

**Primer**	**Sequence (5′-3′)**
4081F	CAA GAC CAC CGG CAA ACC CT
4083R	ATC GAG GCC GTT CGT GCT G
4082_conjunction_F	TCC AAG TCC TTG CCC GGC GTG TCA CTG CGC TAC GAC TTC TGA
4082_conjuction_R	TCA GAA GTC GTA GCG CAG TGA CAC GCC GGG CAA GGA CTT GGA

**Table 3:** **Strains and plasmids used for in-frame deletion of PputGB1_4082 in this study**.

**Strain of plasmid**	**Genotype, characteristics, or construction**	**Source or references**
**STRAINS**		
*E. coli* TAM1	*mcrA* _(*mrr-hsdRMS-mcrBC) _80lacZ_M15 _lacX74 recA1 araD139* (*ara-leu*)*7697 galU galK rpsL endA1 nupG*	Active motif
***P. putida***		
GB-1	Wild type	Corstjens et al., 1992
KG163	PputGB1_4083::pKG220, Gm^R^	Parker et al., 2014
	GB-1 ΔPputGB1_4082	This study
**PLASMIDS**		
pEX18Gm	Gene replacement vector; Gm^r^; *oriT sacB*	Hoang et al., 1998
pJET1.2/blunt	Commercial cloning vector	Fermentas
pEX18Gm_4082	Plasmid for deleting 4082	This study

### Effect of exogenous PVDs on growth of the ferri-PVD receptor mutant of strain GB-1

The ferri-PVD receptor mutant described in the preceding section was inoculated into succinate medium supplemented with 2 μM FeCl_3_. Portions of this culture were supplemented to 10 μM with various PVDs collected from *P. putida* GB-1, *P. putida* KT2440, or *Pseudomonas* sp. PCP1 and grown for ~ 40 h at 220 rpm and room temperature. The effect of each exogenous PVD on the growth of the ferri-PVD receptor mutant was visually observed.

### Effect of exogenous PVDs on biofilm formation by *p. putida* GB-1

To assess the effect of exogenous PVDs on biofilm formation by *P. putida* GB-1, crystal violet staining was used (Yamaguchi, 2009). Briefly, *P. putida* GB-1 were grown in MSGFeMn medium (Parker et al., 2007) for 1 day, then OD_600_ was adjusted to ~0.1 in MSGFeMn media in a series of microtiter plate wells. Each well of 200 μl of this culture was amended with 10 μM PVD from varying *Pseudomonas* species. After 2 days of static incubation, cultures were discarded and the wells were washed with 300 μl PBS three times, air dried, supplemented with 150 μl crystal violet and incubated for 15 min. The plate was then washed under running water, air dried, and 150 μl ethanol (95%) was added to each well. After 30 min of incubation the absorbance was measured at 570 nm using SpectraMax M2 scanning spectrophotometer-fluorimeter (Molecular Devices).

### Effects of exogenous PVDs on PVD production by *p. putida* GB-1

*P. putida* GB-1 was inoculated by diluting a loopful of cells into 1 mL of succinate medium and transferring 10 μL–5 mL of succinate medium amended with 2 μM FeCl_3_ and 10 μM various PVDs collected from different strains. To assess the effects of the varying concentration of PVD, 2 μM Fe(II) was achieved using either FeSO_4_•7H_2_O or FeCl_3_ with 0–10 μM PVD. To determine the effects of varying concentrations of Fe(III) between 0 and 15 μM, FeCl_3_ was added with 10 μM PVD. In all experiments, PVD was added prior to supplementing the media with Fe to enhance the formation of Fe-PVD complexes. PVDs were collected using methods described above from previously siderotyped cultures (Parker et al., 2014). The PVD amended cultures were shaken at 150 rpm and room temperature for 2 days. After 2 days, cells were centrifuged at 13,000 g for 5 min and the supernatant was measured for PVDs. The amount of PVD was estimated by measuring the absorbance at 400 nm (ε = 19,000 M^−1^·cm^−1^) (Parker et al., 2004). All glassware used was soaked in an acid bath for at least 2 days and then rinsed 6–7 times with MilliQ water.

## Results

### MnO_2_ formation during co-growth of *p. putida* GB-1 with various PVD producers

To examine whether co-growth with a strong PVD producer would inhibit MnO_2_ formation by *P. putida* GB-1, various PVD-producing organisms (“challenge strains”) were streaked in pair-wise combinations with *P. putida* GB-1 on Lept agar plates containing 2 μM FeCl_3_ (Figure 1, Table 1), an iron concentration at which *P. putida* GB-1 was sufficiently iron replete to form MnO_2_ (Figure 1) even though the challenge strains were iron limited enough to synthesize some PVD (Supplementary Information, Figure 1S). Since the inhibitory effect of homologous PVD (PVD_GB-1_) has already been described in liquid cultures supplemented with purified PVD at a variety of growth conditions (Parker et al., 2007), the current experiments can indicate whether similar phenomena occur on solid media and whether other PVD types have the same effect as PVD_GB-1_.

When the challenging PVD producer was *P. putida* CFML90-49, an organism that does not oxidize Mn(II) but is of the same siderotype (n°1) as strain GB-1, MnO_2_ production was inhibited, particularly in strain GB-1 growth zones close to strain CFML90-49 (Figure 1E), which coincided with the areas of PVD fluorescence (Figure 1F). This pattern of inhibition and fluorescence resembled that observed for a solution of purified PVD added to one side of a parallel Petri plate (Figures 1A,B). These results indicate that the inhibition previously reported for liquid media (Parker et al., 2007) is also observed on solid media and that solid media reveal spatially localized effects not easily detected in liquid cultures.

The above experiment was repeated with 6 different challenge strains including strain CFML 90-49 and five other *P. putida* strains that also produced siderotype n°1 PVDs. MnO_2_ formation by strain GB-1 was clearly inhibited in 13 out of the 15 Petri plates examined (Table 1). Thus, there is no indication that the inhibitory phenomenon is limited to the previously-studied PVD_GB-1_ and PVD_MnB1_ but apparently applies more generally within siderotype n°1.

MnO_2_ production by strain GB-1 was next examined during co-cultivation with organisms that produced heterologous PVDs, i.e., siderotypes n°2 and n°4. It was hypothesized that heterologous PVDs would not be utilized by strain GB-1 and therefore would reduce iron availability to *P. putida* GB-1, resulting in decreased growth and inhibited MnO_2_ production. These expectations were upheld for challenge by *P. putida* KT2440 (siderotype n°2), but were contradicted for *Pseudomonas* sp. ISO6 (siderotype n°4) (Table 1). That is, MnO_2_ formation by strain GB-1 was inhibited in the presence of *P. putida* KT2440, but was unaffected by the presence of *Pseudomonas* sp. ISO6 (Table 1). Subsequent sections of this paper investigate why the predictions above were not upheld for siderotype n°4, particularly exploring the possibility that *P. putida* GB-1 (siderotype n°1) might be able to utilize siderotype n°4 PVDs for iron nutrition, as well as the predicted outcomes of that possibility.

### Effect of exogenous PVDs on growth of a PVD synthesis mutant of *p. putida* GB-1

**Figure 2:**
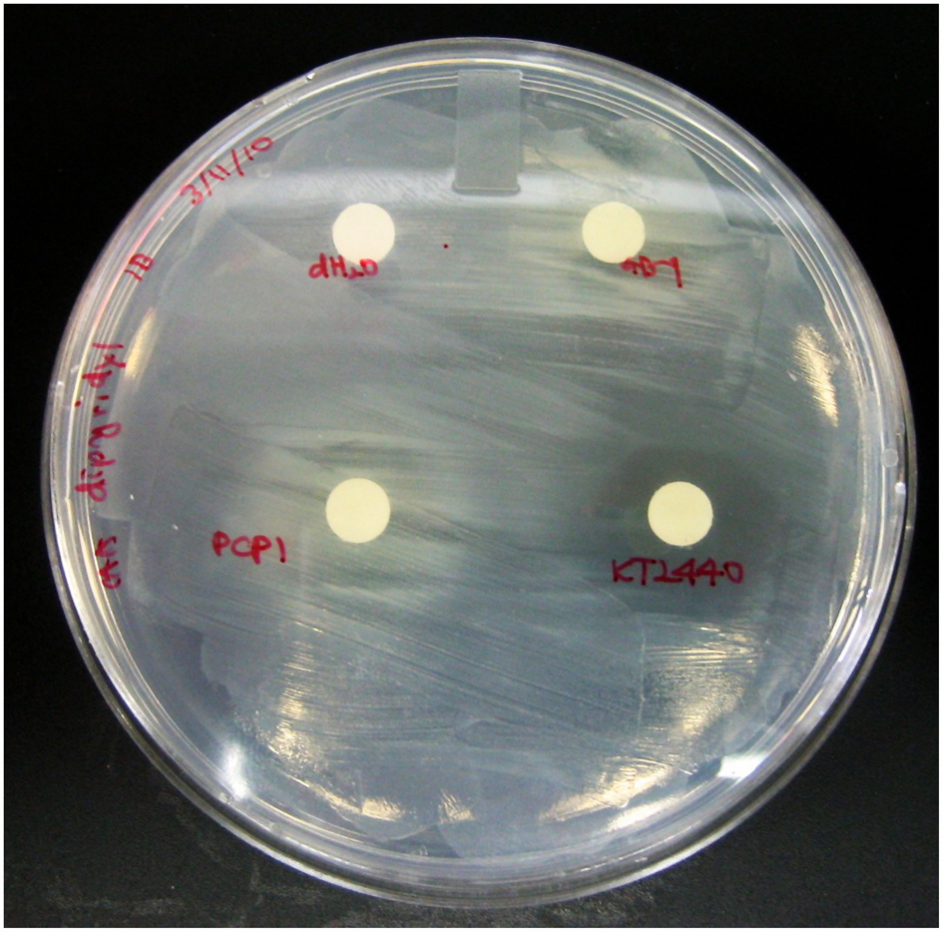
**The *P. putida* GB-1 NRPS mutant KG163, which does not produce PVD, was overlayed on CAA plates supplemented with 100 μM dipyridyl, an agent that strongly complexes Fe**. 20 μl of PVD solutions from *P. putida* GB-1, *P. putida* KT2440, and *Pseudomonas* sp. PCP1 or MilliQ water were put on filter disks to determine the ability of exogenous PVDs to release Fe from it dipyridyl complex and to support the growth (observed as visual turbidity) of a *P. putida* GB-1 mutant that lacks the ability to produce PVD.

To assess whether PVDs of various siderotypes can support the growth of *P. putida* GB-1 (siderotype n°1) in a medium with low iron availability, we utilized liquid cultures of the non-PVD-producing mutant KG163 (PputGB1_4083::pKG220, Gm^R^), in which the NRPS that assembles the PVD backbone has been inactivated by insertion (Parker et al., 2014). Strain KG163 cultures containing the strong Fe(III)-complexing agent dipyridyl were supplemented with PVD solutions representing siderotypes n°1, n°2, and n°4 (Figure 2). As expected, growth was supported by PVD_CFML90−51_ (siderotype n°1), which is indistinguishable from PVD_GB-1_ by several criteria (Parker et al., 2014). In contrast, growth was absent in the presence of the PVD_*KT*2440_ (siderotype n°2), thus showing that *P. putida* GB-1 cannot utilize PVD_KT2440_ for iron uptake. However, growth was observed with PVD_PCP1_ (siderotype n°4), suggesting that strain GB-1 was capable of utilizing this heterologous PVD for growth (Figure 2).

The mechanism through which *P. putida* GB-1 utilizes PVD_PCP1_ to stimulate growth in iron limiting media could involve Fe-PVD_PCP1_ uptake via the receptor for PVD_GB-1_, but it could also involve another receptor or process. To investigate whether the PVD_GB-1_ receptor was required for uptake of PVD_PCP1_ (siderotype n°4), it was first necessary to obtain a mutant deficient in the PVD_GB-1_ receptor.

### In-frame deletion of a gene encoding the putative receptor of PVD_GB-1_

**Figure 3:**
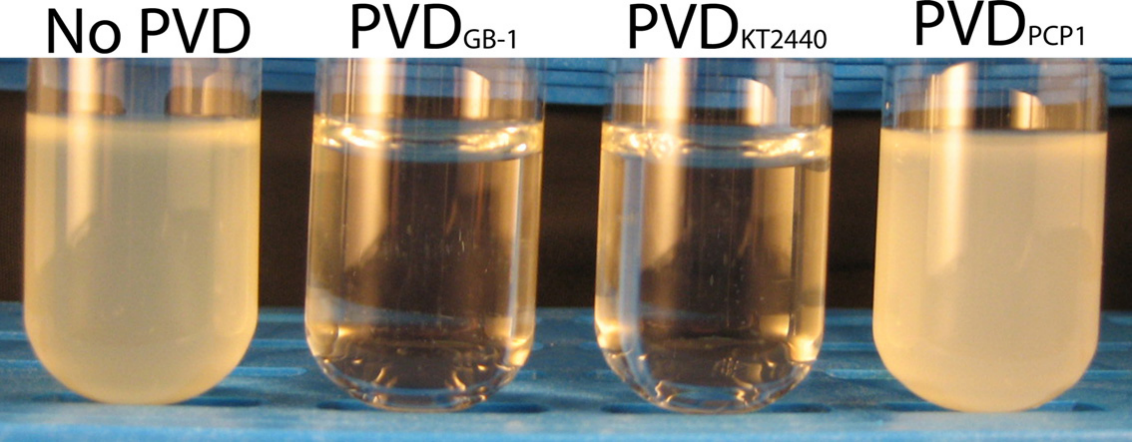
**Effect of exogenous PVDs on the growth of the *P. putida* GB-1 mutant ΔPputGB1_4082**. The medium was supplemented with 2 μM FeCl_3_ and 10 μM of either: (i) no PVDs, (ii) PVD_GB-1_, (iii) PVD_KT2440_, or (iv) PVD_PCP1_.

In the annotation of the genome of *P. putida* GB-1 available on the IMG website, (http://img.jgi.doe.gov/cgi-bin/w/main.cgi), 44 genes encoding putative TonB-dependent receptors were found (McCarthy and Tebo, unpublished data). Out of these genes, PputGB1_4082 was located adjacent to the PVD synthase responsible for producing the peptide backbone of the PVD_GB-1_ (Parker et al., 2014). Since it was expected that PputGB1_4082 might therefore encode the ferri-PVD uptake receptor for PVD_GB-1_, in-frame deletion of PputGB1_4082 was performed. This mutant did not grow when it was cultured in a medium containing 2 μM FeCl_3_ in combination with excess (10 μM) purified PVD_GB-1_ (Figure 3). Since excess PVD can be expected to sequester Fe(III) in PVD complexes that are internalized only by its cognate Fe-PVD receptor, this result confirms that ΔPputGB1_4082 is deficient in the PVD_GB-1_ receptor and also indicates that *P. putida* GB-1 possesses only one cognate receptor, unlike *P. aeruginosa* PAO1, which has two, FpvA and FpvB (Ghysels et al., 2004).

### Effect of exogenous PVDs on growth of the receptor mutant ΔPputGB1_4082

To test whether PVDs not produced by strain GB-1 were accessible to this receptor mutant, cultures of ΔPputGB1_4082 containing 2 μM FeCl_3_ were supplemented with an excess (10 μM) of either PVD_KT2440_ (siderotype n°2) or PVD_PCP1_ (siderotype n°4). PVD_KT2440_ did not support growth of the receptor mutant (Figure 3), just as it had not been accessible to the PVD synthesis mutant (Figure 2), further supporting the interpretation above that Fe-PVD_KT2440_ complexes cannot be detectably used by strain GB-1 via any pathway. In contrast, PVD_PCP1_ supported normal growth of both the receptor mutant (Figure 3) and the PVD synthesis mutant (Figure 2). Therefore, the utilization of Fe-PVD_PCP1_ complexes by strain GB-1 must involve a system that is independent of the cognate (siderotype n°1) PVD receptor (PputGB1_4082).

### Effect of exogenous PVDs on biofilm formation by *p. putida* GB-1

**Figure 4:**
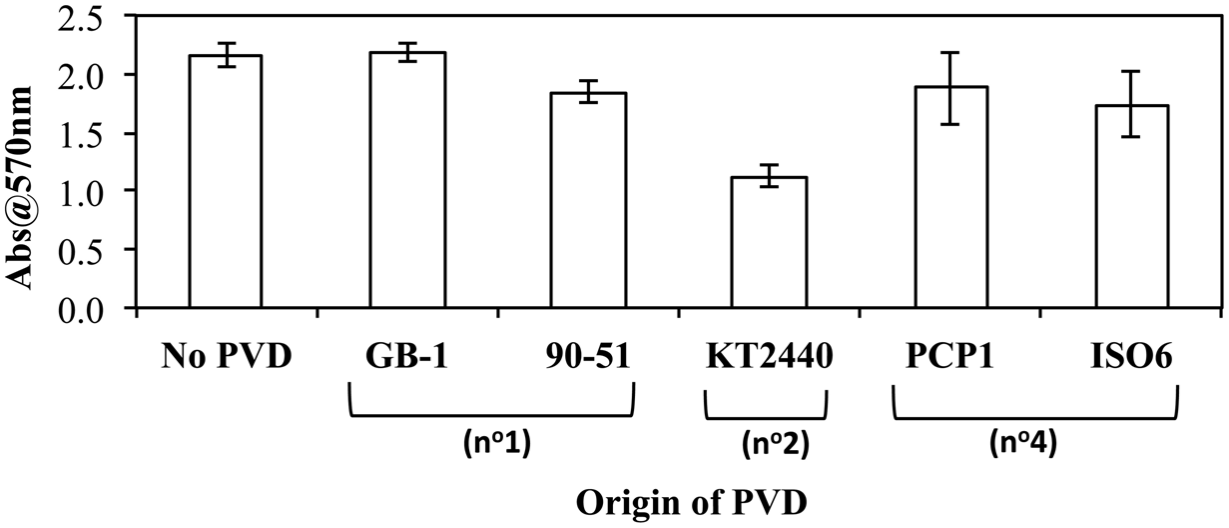
**The effect of exogenous PVDs on biofilm formation by *P. putida* GB-1**. Biofilm formation was estimated using crystal violet staining. [Fe(III)] = 2 μM (added as FeCl_3_) and [PVD] = 10 μM (if added). Error bars indicate the standard deviation of measurements of triplicate samples.

As iron metabolism can affect biofilm formation (Banin et al., 2005), the effect of exogenous PVDs on biofilm formation by *P. putida* GB-1 was tested. It was hypothesized that if the PVD could be utilized by *P. putida* GB-1 then biofilm formation would not be affected, whereas the sequestration of Fe within a non-utilizable PVD complex would inhibit biofilm production. Only PVD_KT2440_ (siderotype n°2) inhibited biofilm formation by *P. putida* GB-1, with the effect statistically significant at the 99% confidence interval (Figure 4), although two siderotype n°1 PVDs (PVD_GB-1_ and PVD_CFML90−51_) and two siderotype n°4 PVDs (PVD_PCP1_ and PVD_ISO6_) did not (Figure 4). This result supports the data in preceding sections, suggesting that only PVD_KT2440_ (siderotype n°2) sequesters Fe(III) in a form that is inaccessible to strain GB-1, whereas the iron complexes of siderotype n°1 and n°4 PVDs can be utilized by this strain.

### Effect of exogenous PVDs on PVD synthesis by *p. putida* GB-1

**Figure 5:**
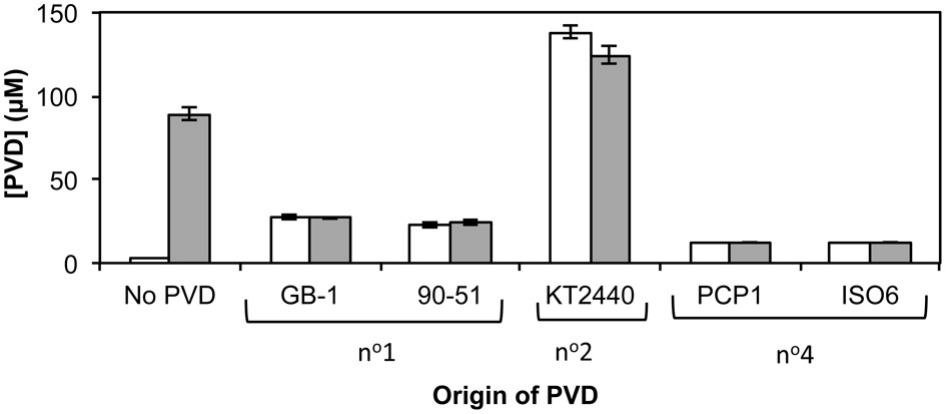
**Concentration of PVD observed 2 days after the addition of various exogenous PVDs to *P. putida* GB-1 cultures**. [Fe] = 2 μM [added as FeCl_3_ (dark bars) or FeSO_4_•7H_2_O (light bars)] and added [PVD] = 10 μM (if added). The exogenous PVDs were: siderotype n°1, PVD_GB-1_ and PVD_CFML90−15_; siderotype n°2, PVD_KT2440_; siderotype n°4, PVD_PCP1_ and PVD_ISO6_. Error bars indicate the standard deviation of measurements of triplicate samples.

PVD synthesis by strain GB-1 after challenge with siderotype n°1, n°2, and n°4 PVDs was also tested as an indicator of iron stress (Figure 5), with PVD concentrations being measured after 48 h of growth in triplicate cultures supplemented at 0 h with 2 μM FeCl_3_ and a 5-fold excess of each tested PVD (Figure 5). These results support the conclusions above concerning the action of each PVD siderotype. Specifically, the addition of the non-utilizable PVD_KT2440_ (siderotype n°2) stimulated PVD synthesis, whereas the addition of utilizable PVDs representing siderotype n°1 (PVD_GB-1_ or PVD_CFML90−51_) or siderotype n°4 (PVD_PCP1_ or PVD_ISO6_) substantially reduced the final PVD concentration, in comparison to the control without added PVD (Figure 5).

**Figure 6:**
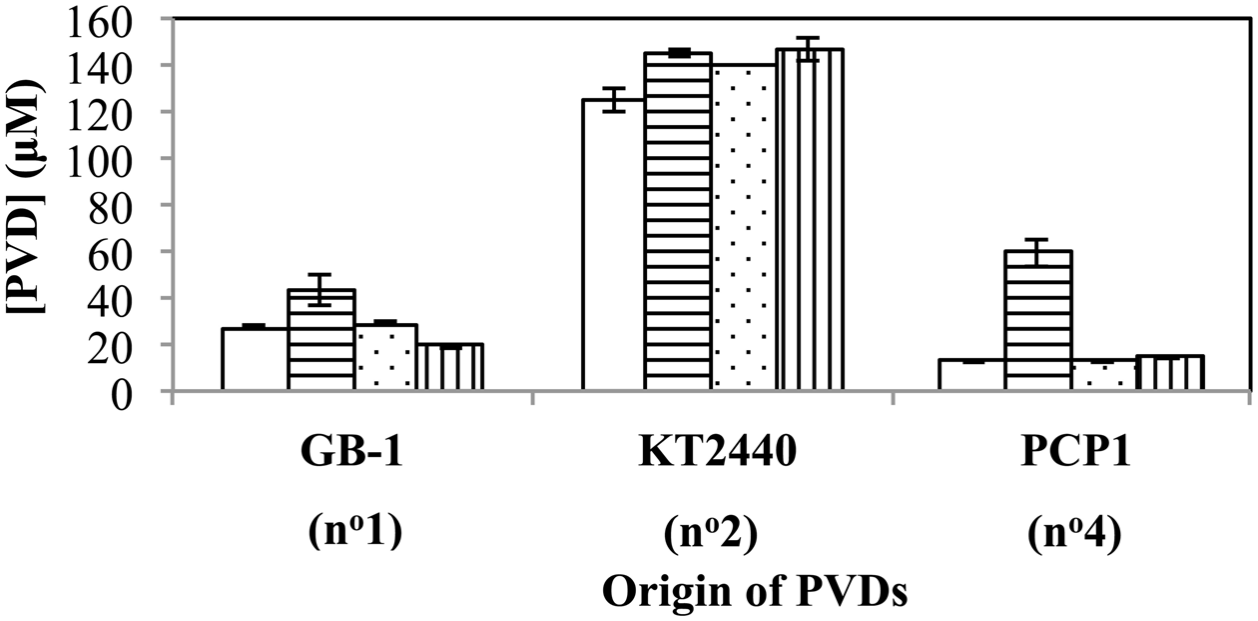
**Concentration of PVD present 2 days after the addition of various exogenous PVDs to each of four different siderotype n°1-producing strains: *P. putida* GB-1 (open), *P. putida* CFML90-49 (horizontal lines), *P. putida* MnB1 (dots), and *P. putida* BC (vertical lines)**. [Fe(III)] = 2 μM (added as FeCl_3_) and added [PVD] = 10 μM. Error bars indicate the standard deviation of triplicate samples.

The above relationships also held true when several additional siderotype n°1 organisms (*P. putida* strains CFML90-51, MnB1 and BC) were challenged with PVD_GB-1_, PVD_KT2440_ and PVD_PCP1_ (Figure 6). The one exception was that *P. putida* CFML90-51 synthesized considerably more PVD in the presence of PVD_PCP1_ than did the other strains (Figure 6), raising the possibility that strain CFML90-51 might utilize siderotype n°4 PVDs much less efficiently, if at all, in comparison to the three other siderotype n°1 organisms tested, strains GB-1, MnB1, and BC. This conclusion is consistent with the previous report that *P. putida* CFML90-51 cannot efficiently incorporate ^59^Fe-labeled PVD_PCP1_ when this property was measured as a part of the initial siderotyping of strain PCP1 and its PVD (Parker et al., 2014). Thus, the ability to utilize siderotype n°4 PVDs may be limited to some, but not all, siderotype n°1 strains of *P. putida*. Such a difference, which perhaps involves the acquisition of additional receptor(s) for certain heterologous PVDs, is not too surprising because strain CFML90-51 comes from a very different habitat and location from the other strains: strain CFML90-51 is a clinical isolate from France (Meyer et al., 2007) whereas the other strains are derived from freshwater habitats in the United States (Francis and Tebo, 2001).

Whether the initial form of iron, i.e., Fe(II) vs. Fe(III), influences PVD synthesis with added exogenous PVD was also tested. As PVD can complex Fe(II) to form Fe(II)-PVD which air oxidizes to Fe(III)-PVD (Xiao and Kisaalita, 1998), it was hypothesized that in the presence of PVD, PVD synthesis would be independent of the form of iron used. This hypothesis was upheld as shown in Figure 5, where PVD synthesis by *P. putida* GB-1 in the presence of exogenous PVDs did not differ when the initial form of iron was changed. However, in the absence of added PVD, Fe(II) could be directly utilized by the cells and therefore PVD synthesis was repressed.

### Effect of the ratio of exogenous PVD_GB-1_ to Fe on PVD synthesis

An initially puzzling result in Figure 5 was that various homologous (siderotype n°1) PVDs only partially inhibited PVD synthesis in strain GB-1 (final PVD concentration = 27 ± 0.8 μM), whereas the heterologous siderotype n°4 PVDs almost completely suppressed PVD synthesis by *P. putida* GB-1 (final PVD concentration = 13 ± 0.1 μM, similar to the concentration of added PVD, 10 μM). This observation might hint toward a phenomenon reported in *P. aeruginosa* PAO1, for which the interaction of homologous PVD-Fe complex with its cognate receptor initiates a signaling pathway that up-regulates PVD synthesis (Lamont et al., 2002). To explore this idea, we cultured strain GB-1 in a medium containing 2 μM FeSO_4_•7H_2_O because Fe(II) at this concentration does not induce PVD synthesis in the absence of exogenous PVD, whereas Fe(III) does (Figure 5). In the presence of exogenous PVD, however, Fe(II) and Fe(III) have similar effects on PVD synthesis as also shown in Figure 5. Thus, the addition of Fe(II) rather than Fe(III) provided continuing iron nutrition and eliminated any effects arising from intracellular iron limitation in control samples without added PVD.

**Figure 7:**
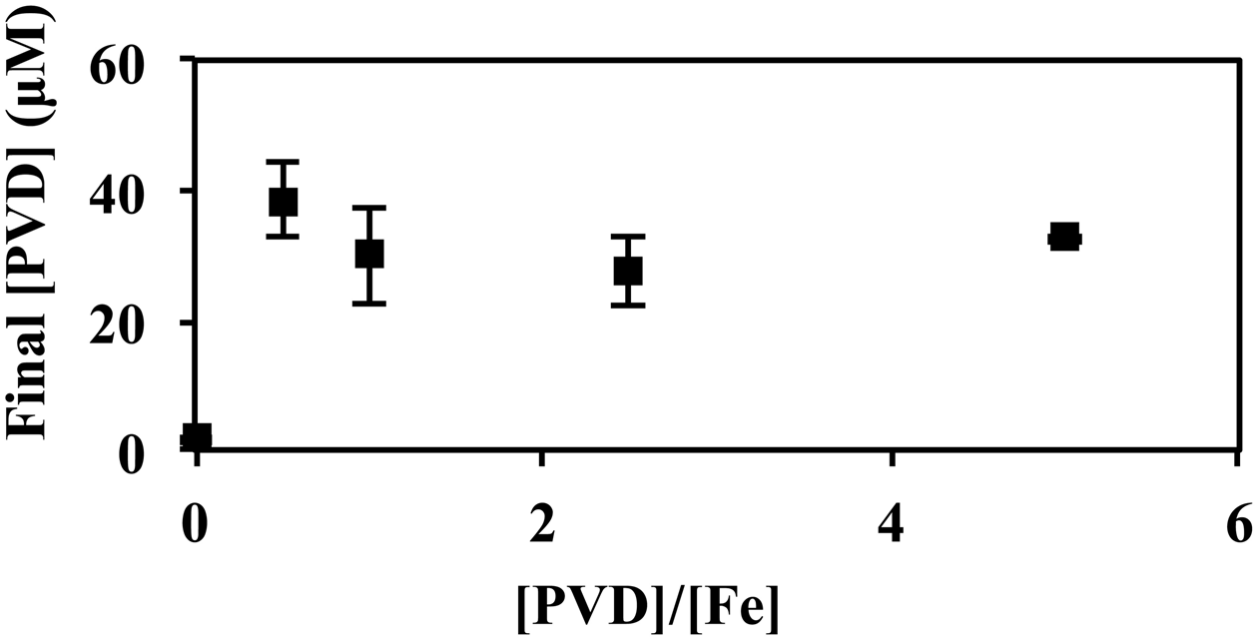
**Concentration of PVD observed 2 days after the addition of varying amounts of PVD_GB-1_ to *P. putida* GB-1 cultures**. [Fe(II)] = 2 μM (added as FeSO_4_•7H_2_O). The concentration of the added PVD was varied from 0 to 10 μM. Error bars indicate the standard deviation of measurement of triplicate samples. Fe(II) was added to fully repress PVD synthesis at the condition without added PVD. For all samples containing PVD, however, the initial Fe(II)-PVD complex was expected to be rapidly air oxidized to Fe(III)-PVD (Xiao and Kisaalita, 1998), consistent with data in Figure 5.

In Figure 7, *P. putida* GB-1 cultures growing on Fe(II) were exposed to PVD_GB-1_ at varying PVD/Fe ratios. In the absence of added PVD, PVD synthesis was negligible supporting the results in Figure 5. Of particular interest were the cultures with a PVD/Fe ratio of 0.5 (2 μM Fe(II) and 1 μM PVD_GB-1_), corresponding to an initial excess of unbound Fe of 1 μM, which is ordinarily enough Fe(II) to repress PVD synthesis in the absence of exogenous PVD at these conditions. Nonetheless, these cultures exhibited considerable PVD synthesis (Figure 7) with a final PVD concentration of 39 ± 6.1 μM roughly comparable to what was observed earlier with homologous PVD (Figures 5, 6) and more than what was observed with siderotype n°4 PVD (13 μM) in Figure 5. Taken together, the results in Figure 7 suggest that the addition of homologous PVD stimulated a moderate amount of PVD synthesis in *P. putida* GB-1. Furthermore, the concentration of synthesized PVD appeared more closely related to the concentration of PVD-Fe complex than the concentration of total PVD or the PVD/Fe ratio (Figure 7).

### Effect on PVD synthesis of the PVD/Fe ratio from exogenous PVDs of various siderotypes

**Figure 8:**
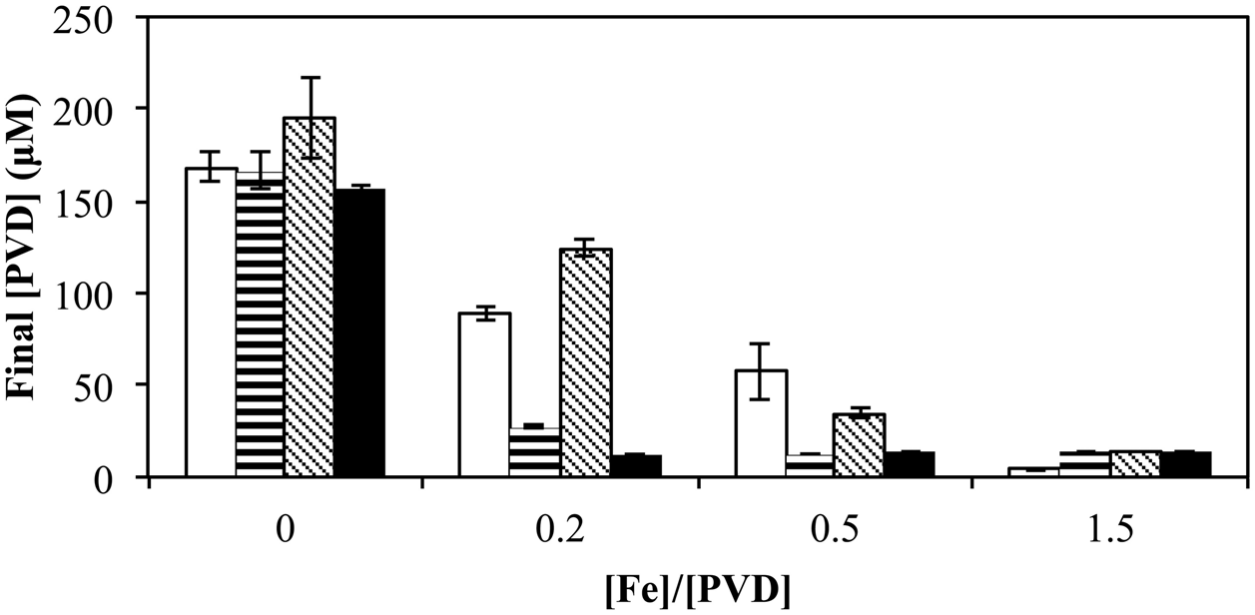
**Concentration of PVD observed 2 days after the addition of varying concentrations of FeCl_3_ to *P. putida* GB-1 cultures supplemented with 10 μM of each indicated PVD**. No PVD (open bars), PVD_GB-1_ (horizontal lines), PVD_KT2440_ (diagonal lines), and PVD_PCP1_ (filled). [Fe(III)] = 0, 2, 5, and 15 μM (added as FeCl_3_) and added [PVD] = 10 μM. Error bars indicate the standard deviation of PVD assays of triplicate samples.

When the total [Fe] was varied (0–15 μM) with a fixed amount of various exogenous PVDs (10 μM), the presence of a utilizable PVD (PVD_GB-1_ or PVD_PCP1_) increased the degree of repression at the intermediate FeCl_3_ concentrations whereas the presence of non-utilizable PVD_KT2440_ had the opposite effect (Figure 8), as would be expected if the utilizable PVDs provided another pathway of Fe uptake in addition to those available to FeCl_3_ alone, whereas the non-utilizable PVD decreased the concentration of bioavailable Fe. When the exogenous PVD was PVD_PCP1_ the final amount of PVD was the same for all Fe/PVD_PCP1_ ratios between 0.2 and 1.5 (Figure 8). In contrast, this degree of repressed PVD synthesis was not seen with exogenous PVD_GB-1_ until the Fe/PVD_GB-1_ ratio was 0.5 (5 μM FeCl_3_ with 10 μM PVD_GB-1_), consistent with results in Figure 5 showing more residual PVD synthesis in the presence of siderotype n°1 than siderotype n°4 PVDs.

## Discussion

Inhibition by pyoverdine-type (PVD) siderophores of the formation of MnO_2_ has been previously demonstrated for the closely related bacterial strains *P. putida* GB-1 and MnB1 challenged by their own PVDs (Parker et al., 2004), including purified PVD solutions (Parker et al., 2007). In this paper we have extended those studies to PVDs produced by other pseudomonads of various siderotypes.

Siderotyping examines the chemical similarities among PVDs produced by different pseudomonads, places the PVDs into groups of similar or identical structure and predicts which PVDs can substitute for each other in cellular utilization (Fuchs et al., 2001; Meyer et al., 2002a). Ordinarily, one expects that an organism will be able to utilize only those PVDs of the same siderotype that it produces and not those of other siderotypes. However, our results indicate that this prediction does not hold for *P. putida* GB-1. Instead, multiple lines of evidence indicated that strain GB-1, which produces siderotype n°1 PVDs, could also utilize siderotype n°4 PVDs for growth, iron nutrition, and the repression of PVD synthesis (Figures 2–5). Certain other pseudomonads have been reported either to synthesize additional PVD receptors that differ from the homologous PVD receptor (Matthijs et al., 2009; Hartney et al., 2012) or to produce a receptor that recognizes more than one PVD (Meyer et al., 1999, 2002b); other iron uptake mechanisms such as Fe(III) exchange (Stintzi et al., 2000) or reduction (Kranzler et al., 2011) are also possible. In the case of strain GB-1, its homologous PVD receptor that recognizes PVD_GB-1_ (siderotype n°1) is not required for the utilization of PVD_PCP1_ (siderotype n°4) because in-frame deletion of that receptor in the mutant ΔPputGB-1_4082 did not block the ability of PVD_PCP1_ to support growth (Figure 3). Furthermore, general mechanisms like Fe(III) exchange or reduction, which can be expected to affect all foreign PVDs fairly equally, are unlikely explanations of siderotype n°4 utilization because strain GB-1 could not utilize a siderotype n°2 PVD (Figures 2–5). Multiple genes encoding putative TonB-dependent receptors are present in the *P. putida* GB-1 genome, including some that might be responsible for the uptake of various siderophores (McCarthy and Tebo, unpublished). Therefore, as in other pseudomonads such as *P. fluorescens* Pf-5 or *P. entomophila* L48 (Matthijs et al., 2009; Hartney et al., 2012), *P. putida* GB-1 has the potential to utilize heterologous siderophores via receptors other than its homologous PVD receptor (Cornelis and Matthijs, 2002).

The Fe-PVD complexes of various PVDs, each added as the sole Fe source, were tested for their ability to support the growth of wild type *P. putida* GB-1 and two of its mutants: strain KG163, which has been shown not to synthesize PVD and to be defective in the NRPS that assembles the backbone of PVD_GB-1_ (Parker et al., 2014) and the mutant ΔPputGB-1_4082, which we here demonstrate to be defective in the uptake receptor for PVD_GB-1_. The results (Figures 2, 3) indicate that representatives of the three tested PVD siderotypes comprise three distinct iron uptake groups. That is, Fe-PVD_GB-1_ (siderotype n°1) supports the growth of strain wild type GB-1 and requires PputGB-1_4082 for its uptake because no growth is observed for the mutant ΔPputGB-1_4082. Fe-PVD_KT2440_ (siderotype n°2) is not detectably utilized by wild type or any of the mutants and thus cannot be incorporated via the PputGB-1_4082 receptor or any other process active at our conditions. In contrast, Fe-PVD_PCP1_ (siderotype n°4) supports the growth of the wild type and both mutants, including ΔPputGB-1_4082, and thus does not require this receptor for its incorporation. The differing uptake patterns of these three Fe-PVD complexes establish the possibility of distinguishing between metabolic phenomena that require participation of the PVD_GB-1_ receptor (PputGB1_4082) vs. those that depend on simple iron nutrition without obligatory involvement of that receptor.

As explained above, the Fe complexes of siderotype n°2 and n°4 PVDs do not interact with the PputGB1_4082 receptor and thus can be used to explore phenomena not involving that receptor. Among all tested Fe-PVD complexes of these two groups, those that repress PVD synthesis by *P. putida* GB-1, suggesting iron sufficiency, also support biofilm and MnO_2_ production. In contrast, those that strongly stimulate PVD synthesis, suggesting iron limitation, can not support biofilm or MnO_2_ production. These data imply a role of iron availability in both biofilm synthesis and Mn(II) oxidation by strain GB-1.

For siderotype n°1 PVDs, which do interact with the PputGB-1_4082 receptor, the situation is more complicated than that of the two siderotypes of heterologous PVDs. For example, the partial induction of PVD synthesis in the presence of added siderotype n°1 PVDs (Figures 5, 7, and 8) implies involvement of this receptor, a phenomenon that may be related to a mechanism reported for *P. aeruginosa* PAO1, in which the interaction of homologous Fe-PVD with its cognate receptor signals PVD synthesis (Lamont et al., 2002). Some support for this idea comes from the observed effects of the ratio of added Fe/exogenous PVD_GB-1_ on subsequent PVD synthesis by strain GB-1: the concentration of synthesized PVD seemed to be more strongly related to Fe-PVD_GB-1_ than the PVD_GB-1_ concentration (Figure 7). In any case, the simplest interpretation of the inhibition of MnO_2_ formation by siderotype n°1 but not siderotype n°4 PVDs is that the inhibition by siderotype n°1 PVD involves PputGB1_4082 in some way, since both PVD types can support growth under iron-limited conditions but only siderotype n°1 requires PputGB1_4082 for Fe-PVD uptake. It remains to be determined whether this phenomenon is directly or indirectly related to a signaling of PVD synthesis via the PputGB1_4082 receptor system or to some other regulatory mechanism. In fact, even for the heterologous, non-utilized siderotype n°2 PVD_KT2440_, it is unclear whether iron itself or the absence of homologous PVD synthesis is the proximal factor required for Mn(II) oxidation in *P. putida* GB-1.

An alternative hypothesis to explain PVD effects on MnO_2_ formation is the reductive dissolution of MnO_2_ by PVD, particularly if the oxides contain substantial Mn(III) (Duckworth and Sposito, 2005). This may not be a valid explanation since the average oxidation state of Mn oxides formed by a closely related strain *P. putida* MnB1 is 3.9, indicating that most of the Mn would be Mn(IV) (Villalobos et al., 2003). In any case, siderophore-mediated dissolution of MnO_2_ likely plays only a minor role, if any, for the results described here because the presence of utilizable heterologous PVD (siderotype n°4) did not inhibit the appearance of MnO_2_. If affinity for Mn(III) of siderotype n°4 PVDs is comparable to that of siderotype n°1 PVD (i.e., PVD_GB-1_), dissolution of Mn minerals mediated by PVD_PCP1_ would be at relatively similar levels promoted by PVD_GB-1_. However, the affinity of siderotype n°4 PVD for different Mn oxidation states or minerals have not been specifically investigated, nor has the average oxidation state of the MnO_2_ in our system been measured.

Experiments involving co-growth of the Mn(II)-oxidizer *P. putida* GB-1 with various PVD producers suggest that the type of siderophore produced by a competing organism may be important in influencing Mn and biofilm metabolism in strain GB-1 (Table 1, Supplementary Information, Figure 1S). Principles related to “cheating” and “predation” in siderophore-based competition among environmental organisms (Meyer et al., 2002b; Griffin et al., 2004; Harrison et al., 2008; Harrison and Buckling, 2009) seem applicable to *P. putida* GB-1 and by extension to other fluorescent pseudomonads, which are widely distributed and prevalent bacteria in many habitats. For example, the ability of PVD_PCP1_ to support the growth of strain GB-1 without stimulating its PVD synthesis can be considered an example of how *P. putida* can “cheat” by not wasting resources to produce homologous PVD when a utilizable heterologous PVD is available, thus conserving resources for other metabolic processes. Our studies suggest that situations involving such cheating are likely to influence whether certain metabolic capabilities, such as Mn(II) oxidation or biofilm formation, are actually expressed by the pseudomonads in a specific environment. In particular, we predict that utilizable heterologous siderophores should stimulate Mn(II) oxidation and even partially counteract the inhibitory effect of homologous PVD on Mn(II) oxidation in pseudomonads, whereas non-utilizable heterologous siderophores would be expected to strongly inhibit Mn(II) oxidation.

Effects of iron concentration and its ratio to the external PVD concentration would also influence both the growth of pseudomonads and their Mn(II) oxidation. Thus, effects of non-utilizable PVD_KT2440_ are overcome with increased [Fe] (or [Fe]/[PVD]) while those of the utilizable PVD_PCP1_ are the same with varying [Fe] for [Fe]/[PVD] between 0.2 and 1.5 (Figure 8). The addition of exogenous homologous PVD to GB-1 cultures results in an increase in final [PVD] (Figure 5), perhaps due to signaling of PVD synthesis mediated by homologous Fe-PVD binding to its cognate receptor (Lamont et al., 2002). However, this stimulatory effect is not as large as that observed for the non-utilized heterologous PVD, PVD_KT2440_ (Figure 5). Unlike the case with PVD_KT2440_, the initial concentration of homologous PVD does not affect the final concentration of synthesized PVD, suggesting that the signaling of PVD synthesis induced by homologous PVD is tightly regulated for conservation of resources (Figure 7). All of these factors should be considered in predicting whether a pseudomonad is likely to oxidize Mn, or express other iron-requiring metabolic capabilities like biofilm formation, in a particular iron-limited environment in the presence of competing exogenous siderophores. Siderophores have been detected in various terrestrial and marine environments (Powell et al., 1980; Essen et al., 2006; Mawji et al., 2008; Duckworth et al., 2009). Therefore, some of these siderophores would form Fe-siderophore complexes that may be taken up by a particular Mn(II)-oxidizing bacterium without additional siderophore synthesis and consequently influence Mn oxide formation. In contrast, if new siderophore synthesis is induced due to the presence of a Fe-siderophore complex that a certain group of siderophore-producing organisms cannot utilize, the additional supply of siderophores can promote formation of various metal-siderophore complexes. Such Mn(III) siderophore complexes could contribute to the soluble Mn(III) concentrations found in the suboxic zones of environments like the water column of the Black Sea or sediments of the Lower St. Lawrence Estuary (Trouwborst et al., 2006; Madison et al., 2011).

### Conflict of interest statement

The authors declare that the research was conducted in the absence of any commercial or financial relationships that could be construed as a potential conflict of interest.
