# Supplementary material for: Effects of exogenous pyoverdines on Fe availability and their impacts on Mn(II) oxidation by Pseudomonas putida GB-1
Source: Front Microbiol. 2014 Jun 25;5:301. doi: 10.3389/fmicb.2014.00301 (PMC4070179; doi:10.3389/fmicb.2014.00301)

Supplementary Information

S.-W. Lee *et al.*

Frontiers in Microbiology 89041

MATERIALS AND METHODS

The strains and growth conditions on 2 μM $FeCl_3$-supplemented Leptothrix (Lept) agar plates were those used for the MnO_2 determinations in Table 1 of the main manuscript. Emphasis here is on triplicate control plates that contained each organism cultured alone with varying concentration of $FeCl_3$, i.e., 0, 2 or 20 μM The amount of PVD present at a given area on the plate was estimated from fluorescence at six pre-determined positions along each streak zone of each plate (1, 1.5, and 2 cm from the edge of the plate at each end of each streak line). After 4 days of incubation at 23-25 ^oC, black/white TIF images of fluorescence were obtained using the ultraviolet lamp and charge-coupled analog camera of a NucleoVision gel documentation system with GelExpert 3.5 software (NucleoTech Corp., San Mateo, CA) at known integration times of 133-899 msec and settings of f8, maximum brightness, and full field focus. Quantitative analysis was conducted with the Scion Image program (Scion Corp., Frederick, MD) on black/white TIFF files. Relative fluorescence at various locations on Petri plates was normalized to that of an uninoculated plate (subtracted as background) and a purified PVD standard (set at 100 relative fluorescence units), which were photographed in the same frame with each experimental plate. Fluorescence at precise positions was determined using the Scion Image program. For each organism, the averages and standard deviations of fluorescence in 18 streak zone sites (6 per plate) were calculated in Excel (Microsoft Corp.) and converted to per cent of the fluorescence in plates with no added $FeCl_3$.

The above method was confirmed by comparison to red/green/blue JPEG images (2560 x 1920 pixels) of the same plates and standards that were taken at known settings of a Dimage X60 camera (Konica-Minolta), with illumination from a 41 x 22 cm UV transilluminator (UVP, Upland, CA) that was aimed downward at the top of a box (1 m square; 1.2 m high) lined with black velvet. Appropriate color images were converted to black/white TIF files (Microsoft Picture It program) for quantitative analysis with the Scion Image program (Scion Corp., Frederick, MD), but the data were also roughly confirmed by readings with the Dimage Viewer program (Konika-Minolta) on color files.

RESULTS AND DISCUSSION

To assess whether *P. putida* CFML90-51, *P. putida* KT2440, and *Pseudomonas* sp. ISO6, PVD-producing challenge strains used in Table 1, in fact produced PVD under the utilized culture conditions, PVD presence was estimated from the fluorescence at pre-determined positions in each Petri plate. Emphasis is placed here on the fluorescence in control plates that were inoculated with each organism alone, mainly because one can know which strain is producing the PVD fluorescence only when there is just one strain on a Petri plate, although fluorescence was also present on cross-streak plates (D. Parker, unpublished data). Figure 1S shows the response of each organism to varying concentrations of $FeCl_3$ and compares those responses with that of the MnO_2 producer *P. putida* GB-1. In all cases, 20 μM $FeCl_3$ yielded strong inhibition of PVD synthesis at 4 days of growth, whereas 2 μM $FeCl_3$ produced partial repression (Figure 1S). These results are consistent with those in Figure 8 of the main manuscript, which show full repression of PVD_{GB-1} synthesis by 15 μM $FeCl_3$ in liquid cultures, but partial repression with 2

and 5 μM $FeCl_3$. It can be concluded that the three challenge strains produced roughly the expected amounts of PVD in the presence of 2 μM $FeCl_3$ on Lept agar plates.

FIGURE LEGEND

Figure 1S. Effect of Fe(III) concentration on fluorescence intensity at 4 days of incubation, for each organism cultured individually on triplicate Lept agar plates containing 0, 2 or 20 μM $FeCl_3$. Fluorescence was measured at six precise positions on each photographed plate. *P. putida* CFML90-51, green line; *Pseudomonas* sp. ISO6, gold line; *P. putida* KT2440, gray line; *P. putida* GB-1, orange line. Error bars reflect standard deviations.

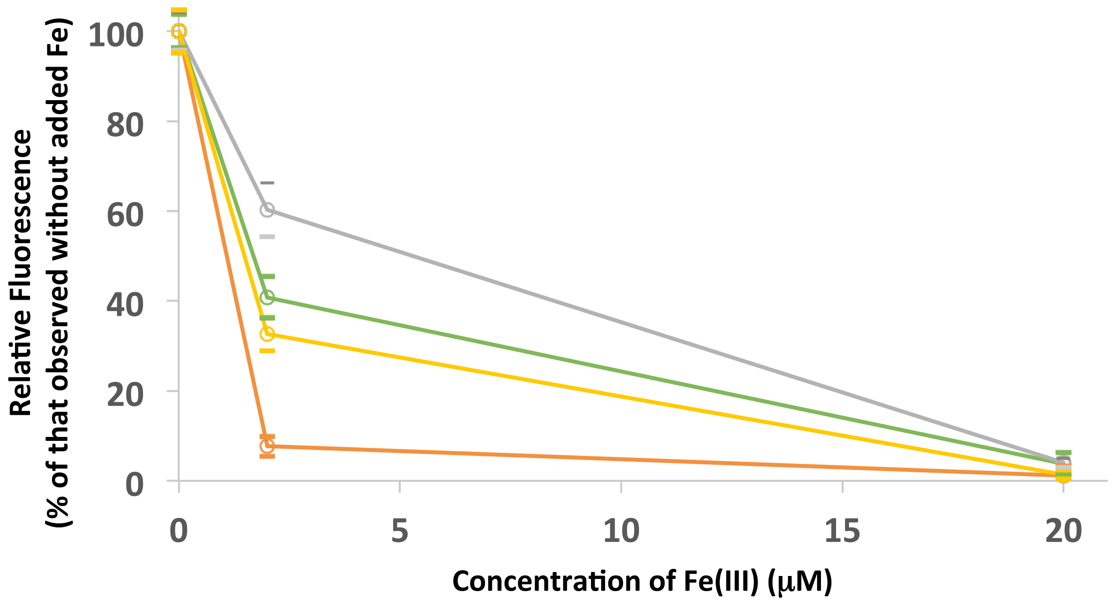

Supplement: Supplementary file 1 [file DataSheet1.PDF]
